# Evaluation of Predictive Factors for Transarterial Bleomycin–Lipiodol Embolization Success in Treating Giant Hepatic Hemangiomas

**DOI:** 10.3390/cancers17010042

**Published:** 2024-12-26

**Authors:** Arkadiusz Kacała, Mateusz Dorochowicz, Adrian Korbecki, Michał Sobański, Agata Zdanowicz-Ratajczak, Dariusz Patrzałek, Dariusz Janczak, Maciej Guziński

**Affiliations:** 1 Department of General, Interventional and Neuroradiology, Wroclaw Medical University, 50-367 Wrocław, Poland; 2 Department of Vascular, General and Transplantation Surgery, Wroclaw Medical University, 50-367 Wrocław, Poland

**Keywords:** giant hepatic hemangiomas, transarterial bleomycin–lipiodol embolization, bleomycin, lipiodol, treatment response, predictive factors

## Abstract

**Introduction:** Giant hepatic hemangiomas are challenging to manage, requiring effective therapeutic approaches. Transarterial bleomycin–lipiodol embolization (TACE) has shown promise as a treatment option, yet predictive factors for its success are not well defined. This study aimed to assess the efficacy of TACE for giant hepatic hemangiomas and identify factors influencing treatment outcomes. **Methods:** A retrospective analysis of 31 adult patients who underwent TACE with bleomycin and lipiodol between December 2014 and October 2022 was conducted. Clinical parameters including age, sex, hemangioma location, lesion size, bleomycin dose, number of TACE sessions, and follow-up duration were evaluated. The primary outcome was hemangioma volume reduction, with statistical analyses identifying factors associated with significant lesion regression. **Results:** Higher bleomycin doses and longer intervals from procedure to follow-up were positively correlated with hemangioma volume reduction, while variables such as patient sex and lesion location showed no statistically significant impact on clinical success. The findings suggest that increased bleomycin dosage and extended follow-up periods may enhance treatment efficacy. **Conclusions:** The study identifies bleomycin dose and follow-up duration as predictive factors for TACE success in treating giant hepatic hemangiomas, underscoring their role in optimizing therapeutic strategies. These insights contribute to improved treatment personalization for patients with giant hepatic hemangiomas and highlight the need for further prospective studies to validate and expand upon these findings.

## Simple Summary

This study investigates the factors influencing the effectiveness of transarterial bleomycin–lipiodol embolization (TACE) in managing giant hepatic hemangiomas. Through a retrospective analysis of 31 adult patients, the research identifies key factors associated with successful volume reduction post-treatment, such as higher bleomycin doses and extended follow-up periods. Notably, the study found no significant correlation between lesion location or preoperative size and clinical success, highlighting that TACE remains effective across varied lesion sizes. These findings suggest potential for tailored dosing and timing strategies in clinical practice to optimize outcomes, while the study encourages further investigation into refining TACE protocols.

## 1. Introduction

Hepatic hemangiomas, the most prevalent benign mesenchymal liver tumors, have a prevalence ranging from 3 to 20% in autopsy studies [1,2,3]. These tumors primarily affect women and adults in their fourth and fifth decades of life. While hepatic hemangiomas typically do not cause symptoms and individuals maintain normal liver function, they are often incidentally discovered. In the past, surgical resection was the preferred treatment [4]. However, surgical resection may yield suboptimal outcomes and pose a significant risk of bleeding, particularly in patients with multiple lesions or lesions near hepatic portal vessels [5].

Currently, transarterial chemoembolization (TACE) has emerged as a widely accepted treatment for giant hepatic hemangiomas (>5 cm). This procedure offers a compelling blend of minimal invasiveness, low mortality risk, and sustained long-term safety [6,7]. Despite numerous analyses of the safety and efficiency of TACE with varying results [8,9,10,11], there remains a notable gap in the statistical analysis concerning potential predictive factors for the procedure’s safety and efficacy.

Bleomycin is a glycopeptide antibiotic that exerts cytotoxic, antiangiogenic, and sclerosing effects by inducing DNA strand breaks and provoking an inflammatory response in the lesion [12]. When combined with lipiodol—a radiopaque, oil-based contrast agent—the drug can be retained longer in the tumor’s vascular channels, allowing higher local concentrations of bleomycin [13,14]. Hemangiomas, composed of venous lakes with slow blood flow, are particularly amenable to this approach because the viscous nature of lipiodol slows its washout, enhancing bleomycin’s tumoricidal action [15].

Beyond delivering bleomycin, TACE partially occludes hemangioma-feeding arteries. Embolic ischemia further augments tumor necrosis and shrinkage, and hepatic hemangiomas, with their characteristic sluggish blood flow, can be especially responsive [8,9,16]. Through this dual mechanism (localized cytotoxicity from bleomycin plus ischemia from lipiodol), TACE achieves considerable lesion reduction while minimizing systemic toxicity [10,11,17]. Despite the increasing clinical use of TACE, few studies have systematically examined how factors such as bleomycin dosage or tumor coverage might predict a favorable outcome. Herein, we seek to address that gap by analyzing key predictive factors for treatment response in giant hepatic hemangiomas.

This study aimed to address this gap by conducting a retrospective analysis of collected data to evaluate the predictive factors influencing the effectiveness of bleomycin–lipiodol emulsions in TACE for giant hepatic hemangiomas.

## 2. Materials and Methods

### 2.1. Patient Characteristics and Diagnosis

In this single-center retrospective study, 73 patients diagnosed with giant hepatic hemangiomas underwent transarterial chemoembolization with a bleomycin–lipiodol emulsion from December 2014 to October 2022. A focused subgroup of 31 patients with available follow-up imaging data was analyzed to identify predictive factors influencing TACE efficacy with the bleomycin–lipiodol emulsion.

Inclusion criteria required patients to have giant hepatic hemangiomas confirmed through imaging (e.g., CT or MRI) with diameters exceeding 5 cm. Comprehensive patient data were collected, including hemangioma size, number, liver involvement, and pre-TACE assessments. Clinical success was defined as a 50% or greater reduction in hemangioma volume on follow-up imaging, and technical success was indicated by successful embolization of all feeding arteries.

After TACE, angiography was used to assess drug coverage along hemangioma borders, measured on a four-point scale to determine drug dispersion. Pain intensity was recorded using a four-point verbal rating scale. Patients were closely monitored for adverse events, with mortality rates assessed during the procedure and hospital stay.

Hemangioma volume reduction was evaluated by CT or MRI approximately 12.5 ± 11.3 months after the procedure. When necessary, additional TACE sessions were considered based on the degree of volume reduction or symptom recurrence. This targeted approach aimed to identify predictive factors for the efficacy of bleomycin–lipiodol emulsion in TACE for giant hepatic hemangiomas.

### 2.2. Treatment

Under local anesthesia, an 18 G needle was used to puncture the common femoral artery, and a 5F femoral sheath was inserted. A 5F Simon 1 or Cobra 2 catheter was used to navigate the celiac trunk and superior mesenteric artery. Arteriography then identified the primary feeding artery of the hemangioma, after which a 2.4Fr or 2.7Fr microcatheter was guided into the feeding artery. Under fluoroscopic guidance, a solution containing 1500 IU of bleomycin (Pfizer Inc., New York, NY, USA) and 7–15 cc of lipiodol (Guerbet, Villepinte, France) was injected into the hemangioma using standard three-way stopcocks until optimal distribution was achieved. The maximum bleomycin dose per session was 15,000 IU, and the lipiodol dose was adjusted to the hemangioma’s size, not exceeding 30 mL per session.

### 2.3. Predictive Factors

The study conducted a comprehensive evaluation of all relevant patient data to identify factors affecting the likelihood of achieving clinical success. This analysis focused on the influence of various patient characteristics, aiming to explore the validity of TACE across diverse patient groups and investigate the potential for tailoring treatment approaches based on individual characteristics.

Additionally, intraoperative factors, such as bleomycin dosage and its potential impact on procedural effectiveness, were meticulously examined. These findings could lead to a reassessment of bleomycin dosing guidelines to optimize patient outcomes. The coverage of hemangioma borders was carefully analyzed for its correlation with lesion volume reduction, given lipiodol’s critical role as a drug carrier within the target area.

The study also investigated the time interval between the procedure and follow-up, seeking to establish an optimal duration for lesion shrinkage. This analysis facilitated a reassessment of the timing between initial and subsequent interventions. Moreover, the relationship between volume reduction and the number of procedures was validated, supporting additional interventions based on individual patient responses.

The study extended its analysis to factors influencing the incidence and severity of post-embolization syndrome (PES). Specifically, the relationship between bleomycin dosage and PES severity was explored, as tissue ischemia due to bleomycin administration is a primary cause of PES. The role of lipiodol in contributing to tissue ischemia was also analyzed in the context of PES. Finally, the correlation between hemangioma border coverage and PES severity was examined.

In summary, this thorough methodology allowed for a comprehensive analysis of predictive factors within the study, offering insights into potential strategies for refining treatment approaches and enhancing patient outcomes.

### 2.4. Statistical Analysis

Statistical analysis was performed using R software (version 4.1.2). Nominal variables were expressed as counts and percentages, while numeric variables were summarized as mean (SD) or median (IQR), based on distribution normality assessed by the Shapiro–Wilk test, skewness, and kurtosis. Variance homogeneity was checked using the Levene test. Group comparisons utilized t-Student, t-Welch, or Mann–Whitney U tests for numeric variables, and chi-squared or Fisher exact tests for nominal variables as appropriate. Pearson or Spearman correlation analysis was used to assess relationships between numeric variables, depending on distribution normality.

To identify factors associated with clinical success, a two-step logistic regression was applied. Variable selection for the multivariate model initially used a *p* < 0.25 criterion in univariate regression, followed by a stepwise selection for the final model. The fit of the multivariate model was assessed with Nagelkerke R² and the Hosmer and Lemeshow GOF test, while collinearity was examined using VIF indicators. All statistical tests assumed an alpha level of 0.05.

The analysis compared groups with and without clinical success to uncover significant associations and quantify factors influencing the odds of achieving clinical success through a two-step logistic regression analysis.

## 3. Results

### 3.1. Patients Characteristics

The study comprised 31 patients, with the majority (80.6%) being female. The average age at the first procedure was 49.39 ± 10.93 years, ranging from 30.28 to 69.64 years. All patients reported experiencing varying degrees of abdominal symptoms, including distension, nausea, abdominal pain, dyspepsia, or early satiety. The mean pre-procedure lesion volume measured 241.04 cm^3^, ranging from 51.91 cm^3^ to 2828.8 cm^3^. In terms of embolization, 45.2% of patients underwent one procedure, 32.3% had two procedures, and five and two patients underwent three and four procedures, respectively. All procedures were conducted at a single facility to ensure consistency and standardized patient care. Table 1 presents essential patient characteristics, including age, gender, and pertinent medical history, displaying mean values alongside their respective standard deviations.

### 3.2. Association Among Factors Influencing Clinical Success

A comparative analysis was undertaken to explore the factors associated with clinical success, seeking to identify significant contributors to this outcome. This comparative assessment is presented comprehensively in Table 2, providing a concise summary of the findings.

During the univariate logistic regression analysis, it was observed that none of the examined factors exhibited a statistically significant impact on the likelihood of achieving clinical success. However, a noteworthy trend was observed in relation to gender, with a *p*-value slightly below the conventional significance threshold (*p* = 0.051). This observation hints at a potential association between gender and clinical success, although it does not reach full statistical significance. Specifically, it suggests that there may be a difference in the odds of achieving clinical success between males and females, with males potentially having odds lower by approximately 86% (OR = 0.14) compared to their female counterparts. While not definitive, this finding prompts further exploration and consideration, as it may indicate a gender-related aspect in the context of clinical success that warrants additional investigation.

In the multivariate regression analysis, both sex and the number of embolizations were considered as factors. However, the results indicate that none of these factors exerted a significant influence on the odds of achieving clinical success. It is noteworthy, though, that the variable of sex demonstrated a relationship with clinical success at a significance level just below the conventional threshold (*p* = 0.072). This finding suggests a potential association between gender and clinical success, with males potentially having odds of clinical success that are lower by approximately 92% compared to females, as indicated by an odds ratio (OR) of 0.08.

The reliability of the multivariate model was assessed using Nagelkerke R2, resulting in an outcome of 44.1%, suggesting a reasonably good model fit. Additionally, the Hosmer and Lemeshow goodness-of-fit (GOF) test yielded a *p*-value of 0.307, further supporting the adequacy of the model’s fit. Collinearity was also examined through VIF indicators, which ranged from 1.2 to 1.9, affirming the absence of collinearity issues. The detailed outcomes of the logistic regression analysis pertaining to clinical success are presented in Table 3 for reference.

### 3.3. Association Among Factors Influencing Volume Regression

The potential associations among factors influencing volume regression were assessed. However, statistical analyses did not reveal a significant relationship between volume regression and the coverage of hemangioma borders during the first procedure, with a *p*-value of 0.319. Similarly, the statistical analysis did not establish a significant association between volume regression and the maximal coverage of hemangioma borders (maximum across all procedures per patient), yielding a *p*-value of 0.968. A detailed representation of the relationship between volume regression and the coverage of hemangioma borders, assessed on a 4-point scale, is presented in Table 4.

**Figure 1:**
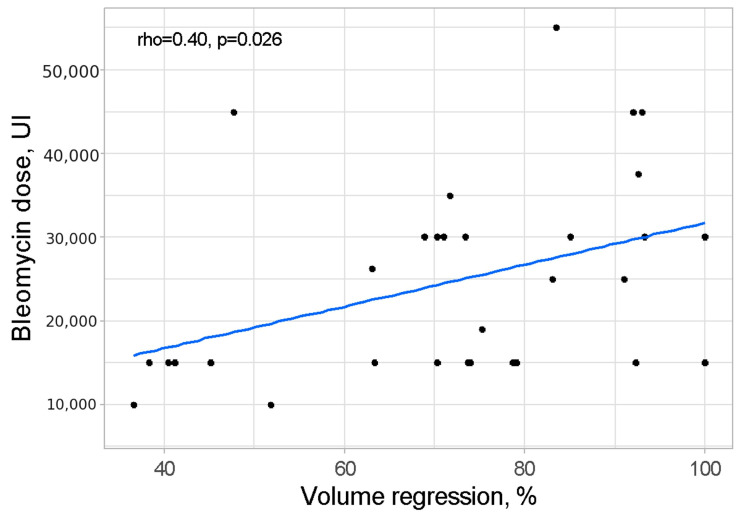
Scatterplot presenting relation between volume regression and bleomycin dose.

The relationship between volume regression (%) and bleomycin dose (IU) was assessed using Pearson correlation analysis, which revealed a statistically significant association with a *p*-value of 0.026. The correlation coefficient indicated a moderate-strength positive relationship (rho = 0.40), signifying that a higher bleomycin dose was linked to a greater degree of volume regression. This relationship is visually depicted in Figure 1.

**Figure 2:**
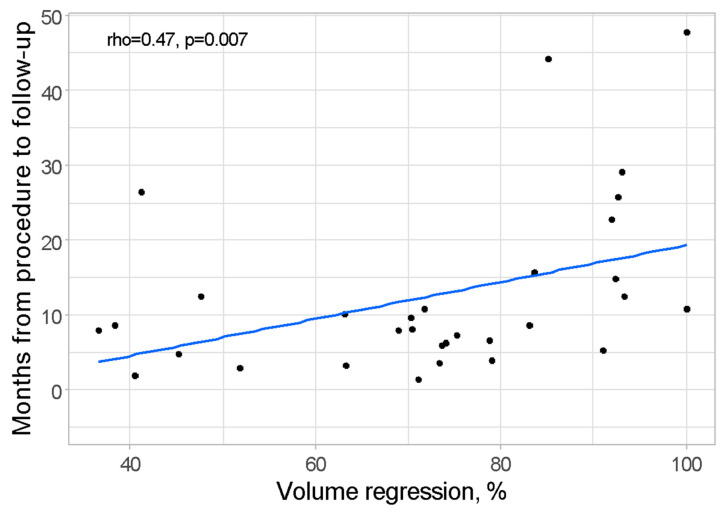
Scatterplot presenting relation between volume regression and time from procedure to follow-up.

The relationship between volume regression (%) and the time elapsed from the procedure to the follow-up (measured in both days and months) was examined through Spearman correlation analysis. The results demonstrated the statistical significance of this relationship, with a *p*-value of 0.007. The correlation coefficient indicated a moderately strong positive association (rho = 0.47), signifying that a longer duration between the procedure and follow-up was correlated with a higher degree of volume regression. A visual representation of this relationship is presented in Figure 2.

**Figure 3:**
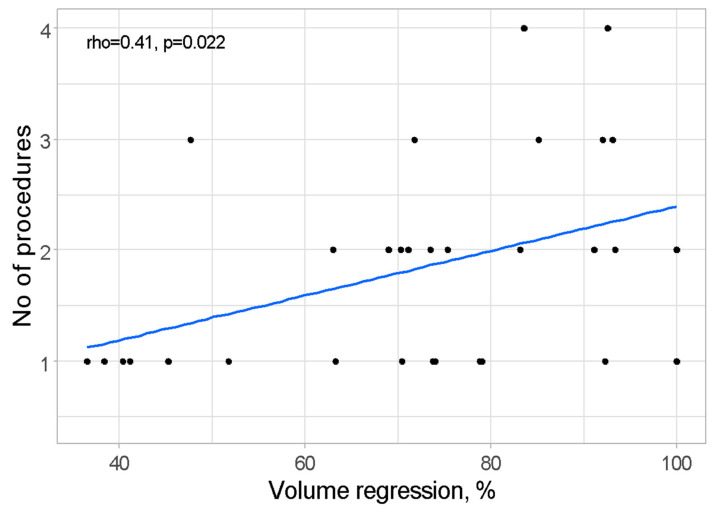
Scatterplot presenting relation between volume regression and number of procedures.

The relationship between volume regression (%) and the number of procedures conducted was assessed through Pearson correlation analysis, revealing statistical significance with a *p*-value of 0.022. The correlation coefficient indicated a moderately strong positive association (rho = 0.41), indicating that a greater number of procedures was linked to more substantial volume regression. This relationship is illustrated in Figure 3.

The statistical analysis also aimed at assessing the relationship between volume regression and the specific location of hepatic hemangiomas within the left or right liver lobe. However, the results of this analysis did not yield statistically significant findings, as evidenced by a *p*-value of 0.986. These findings, which are presented in a formal manner in Table 5, suggest that the location of these hepatic hemangiomas within the liver lobes, whether left or right, does not appear to exert a significant influence on the observed degree of volume regression.

### 3.4. Association Among Factors Influencing the Occurrence and Severity of Post-Embolization Syndrome

The investigation into the association between the severity of post-embolization syndrome and bleomycin dose (IU) utilized Spearman correlation analysis. The results, however, did not reveal statistical significance, with a *p*-value of 0.948. Likewise, the analysis did not confirm a statistically significant relationship between the incidence of vomiting as a component of post-embolization syndrome and bleomycin dose, with a *p*-value of 0.181. These findings are presented in Table 6 for reference.

The investigation into the potential link between the severity of post-embolization syndrome and lipiodol dose for patients who received lipiodol was conducted through Spearman correlation analysis. However, the results did not yield statistical significance, with a *p*-value of 0.674. Similarly, the statistical analysis did not establish a significant relationship between the incidence of vomiting, a component of post-embolization syndrome, and lipiodol dose for patients who were administered lipiodol, as indicated by a *p*-value of 0.418. These findings are visually represented in Table 7 for reference.

The statistical assessment of the potential relationship between post-embolization syndrome and the coverage of hemangioma borders did not yield significant findings. This applied to both the severity of post-embolization syndrome (*p* > 0.999) and the occurrence of vomiting (*p* > 0.999), as detailed in Table 8.

## 4. Discussion

Hepatic hemangiomas—benign vascular liver tumors—are frequently asymptomatic, incidentally discovered on imaging, and seldom compromise liver function [13,14,15,16,17]. Although malignant transformation has not been documented, certain factors such as high estrogen states can spur growth [18,19]. Giant hemangiomas, however, may elicit significant pain, compress adjacent structures, or provoke complications like Budd–Chiari syndrome or spontaneous rupture, the latter carrying a high mortality rate [20,21,22]. While the risk of spontaneous rupture remains low (1–4%), large subcapsular hemangiomas represent a higher-risk subset [23,24,25,26].

**Figure 4:**
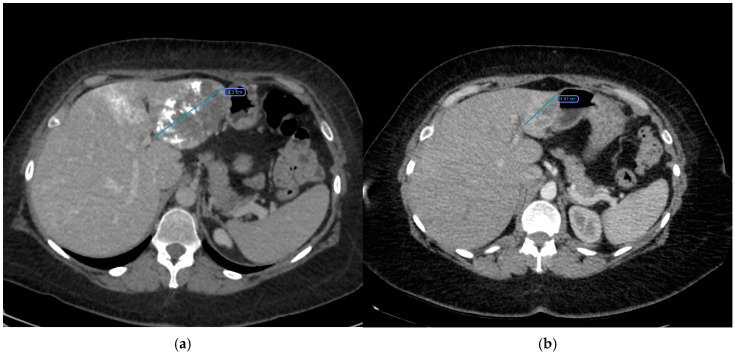
CT scans illustrating a giant hepatic hemangioma before and after treatment: (**a**) CT obtained immediately after the first embolization, showing a hemangioma measuring 8.3 cm at its largest diameter. (**b**) CT obtained 18 months later, demonstrating a significant reduction in hemangioma size (4.2 cm).

Traditionally, symptomatic or enlarging hemangiomas prompted surgical resection [27,28,29,30,31], yet surgery can be complicated by bleeding, extended hospital stays, and morbidity, particularly with large (>10 cm) lesions [32]. Less invasive methods, including radiofrequency (RFA) or microwave (MWA) ablation, have had limited success and higher complication rates in large lesions [33,34,35,36,37]. Consequently, TACE has gained momentum as a minimally invasive treatment, combining bleomycin’s sclerosing effect with lipiodol’s ability to prolong intratumoral drug residence [12,38,39,40] (Figure 4). Despite TACE’s widespread clinical acceptance, evidence concerning predictive factors for treatment success remains limited.

The patient’s sex emerged as a significant factor associated with clinical success in this study. Specifically, males were found to have substantially lower odds of achieving clinical success, with a reduction of 92%. It is important to note that caution must be exercised when interpreting this finding due to the relatively limited number of male patients included in this study, which amounted to only six individuals.

Hepatic hemangiomas are predominantly encountered in women, and, as such, a more extensive study cohort is imperative to draw definitive conclusions regarding the role of sex as a predictive factor for transarterial chemoembolization (TACE) clinical success. Nevertheless, should future investigations corroborate the findings of this study, it may necessitate the development of a more nuanced and personalized approach to the treatment of hepatic hemangiomas, specifically considering the patient’s sex as a potential influential factor.

The absence of lesion location as a statistically significant factor related to clinical success offers valuable insights into therapeutic strategies for hepatic hemangiomas. In traditional surgical approaches, the lesion’s location plays a pivotal role. However, this investigation did not identify any statistically significant differences in this regard. Notably, the study included only one patient diagnosed with a lesion spanning both the right and left liver lobes. Given the rarity of such cases, a substantially larger patient cohort would be necessary to definitively ascertain whether this factor significantly impacts treatment outcomes.

The preoperative volume of hemangiomas was found not to be a statistically significant factor associated with clinical success. In this study, the range of preoperative volumes for the lesions spanned from 51.9 cm^3^ to 2828.8 cm^3^. This wide spectrum of analyzed volumes underscores the suitability of transarterial chemoembolization (TACE) as an effective therapeutic approach for giant hepatic hemangiomas of varying sizes.

The assessment of hemangioma border coverage, conducted through angiography after chemoembolization, is a factor that has received limited attention in previous studies. Past research, notably by Akhlaghpoor et al. [40], has highlighted the correlation between hemangioma border coverage and volume regression. Their findings indicate that an increase in drug coverage grade consistently correlated with a higher percentage of hemangioma shrinkage.

In our study, however, we did not confirm a relationship between volume regression and the coverage of hemangioma borders. It is essential to acknowledge that in our study a coverage rate exceeding 75%, corresponding to grade 4, was achieved in 93.5% of cases. This stands in contrast to the study conducted by Akhlaghpoor et al., where this level of coverage was achieved in only 37.93% of cases. This notable difference in coverage rates resulted in a considerably less diverse study group in our investigation, which may have influenced the observed outcomes.

The bleomycin dose was identified as a statistically significant factor influencing volume regression. The correlation coefficient revealed a moderately strong positive relationship (rho = 0.40), indicating that higher bleomycin doses were associated with more substantial volume regression. It is worth noting that pulmonary fibrosis has been documented as a potential complication in patients exposed to notably high cumulative doses of bleomycin, particularly exceeding 300 mg [41].

Throughout this study, the maximum bleomycin dose administered per session was 15 mg. This finding may suggest the need for a re-evaluation of the bleomycin dosage, given the observed influence on volume regression and the potential risk associated with higher cumulative doses.

The correlation between the time from the procedure to follow-up was previously noted by Yuan et al., who reported a significant interaction between the procedure and time. Their study identified the conclusion of the shrinkage period at the 12-month mark [8].

In this study, the correlation coefficient indicated a moderately strong positive relationship (rho = 0.47). This suggests that a longer duration between the procedure and follow-up was associated with a higher degree of volume regression. However, it is important to note that the limited shrinkage period could not be definitively confirmed due to the absence of periodic follow-ups. Consequently, the shrinkage rate could not be accurately determined, leading to an inability to ascertain the endpoint of the shrinkage period. Nonetheless, this correlation underscores the importance of carefully reassessing indications for additional TACE.

The doses of bleomycin and lipiodol were found not to be significant factors influencing the occurrence and severity of post-embolization syndrome. This underscores the idea that post-embolization syndrome (PES) is primarily attributed to the inflammatory pathophysiology resulting from tumor necrosis and ischemia to healthy liver parenchyma, rather than being linked to the toxic effects of the chemotherapeutic agents.

This study is subject to certain limitations due to its retrospective design. The retrospective nature of the study limited the precise assessment of hemangioma volumes before TACE in the majority of cases. Additionally, the study group was relatively small, particularly concerning rarer factors, which may have affected the statistical analysis. The diagnosis relied on typical imaging features, and biopsy was not performed, as benign lesions like hemangiomas generally do not require biopsy in most cases.

Furthermore, the availability of long-term follow-up data was limited, as asymptomatic patients often did not attend follow-up visits. Consequently, there is a need for further prospective studies with larger study groups and extended follow-up periods to provide a more comprehensive evaluation of the predictive factors for the effectiveness of transarterial bleomycin–lipiodol embolization in patients with giant hepatic hemangiomas.

## 5. Conclusions

This study identified key predictive factors associated with the effectiveness of transarterial bleomycin–lipiodol embolization for the treatment of giant hepatic hemangiomas. Our findings suggest that higher bleomycin doses and extended time intervals from the procedure to follow-up correlate positively with greater lesion volume reduction, indicating that these parameters may enhance treatment outcomes. In contrast, other variables such as patient sex, lesion location, and initial lesion size did not show significant associations with clinical success, underscoring the broad applicability of TACE across various patient profiles and hemangioma characteristics.

The analysis of hemangioma border coverage did not reveal a clear relationship with lesion volume reduction, potentially due to the high degree of coverage achieved in most cases within our cohort. Furthermore, our study found no significant association between bleomycin or lipiodol dosages and the severity of post-embolization syndrome (PES), indicating that PES may be more closely linked to ischemic and inflammatory responses rather than the specific doses of these agents.

These findings contribute valuable insights into the optimization of TACE protocols for giant hepatic hemangiomas, advocating for tailored dosing and follow-up timing to maximize clinical outcomes. However, further prospective studies with larger patient cohorts and longer follow-up periods are essential to validate these predictive factors and refine therapeutic guidelines for TACE. This research lays a foundation for more personalized and effective management strategies, ultimately aiming to improve patient outcomes in the treatment of giant hepatic hemangiomas.

## Figures and Tables

**Table 1:** Characteristics of study observations.

Variable	*n*	Total Group
N	31	
Age (day of 1st procedure), years, mean ± SD	31	49.39 ± 10.93
Sex	31	
Female		25 (80.6)
Male		6 (19.4)
Location	31	
Left liver lobe		13 (41.9)
Right liver lobe		17 (54.8)
Both liver lobes		1 (3.2)
Diagnostics before procedure	31	
TK		21 (67.7)
MR		5 (16.1)
No information		5 (16.1)
Size before procedure, cm		
x, median (IQR)	31	8.70 (7.50;10.55)
y, median (IQR)	31	7.20 (5.50;9.10)
z, median (IQR)	31	7.40 (6.15;9.80)
Volume before procedure, cm^3^, median (IQR)	31	241.04 (123.98;539.08)
Embolization—no of stages	31	
1		14 (45.2)
2		10 (32.3)
3		5 (16.1)
4		2 (6.5)
Embolization—no of stages, mean ± SD	31	1.84 ± 0.93
Bleomycin, UI, mean ± SD	31	24,766.13 ± 11,856.25
Lipiodol, mL, median (IQR)	31	0.00 (0.00;10.00)
Lipiodol > 0, mL, mean ± SD	15	19.60 ± 15.25
Radiation, Gy, median (IQR)	31	0.17 (0.00;0.59)
Radiation > 0, Gy, median (IQR)	17	0.58 (0.25;0.85)
Coverage of hemangioma’s borders *	31	
<25%		1 (3.2)
25–50%		0 (0.0)
50–75%		1 (3.2)
75–100%		29 (93.5)
Complication **	31	3 (9.7)
Post-embolization syndrome—severity, [0–3], median (IQR) ***	31	0.50 (0.00;1.00)
Post-embolization syndrome—severity > 0, [1–3], median (IQR) ***	22	1.00 (0.50;1.25)
Post-embolization syndrome—vomiting	31	5 (16.1)
Hospitalization, days, median (IQR)	31	6.00 (3.00;10.00)
Diagnostics after procedure	31	
TK		29 (93.5)
MR		2 (6.5)
Size after procedure, cm		
x, mean ± SD	31	5.43 ± 2.53
y, median (IQR)	31	4.30 (3.70;5.45)
z, mean ± SD	31	5.19 ± 2.77
Volume after procedure, cm^3^, median (IQR)	31	59.59 (33.75;104.49)
Volume regression, %, mean ± SD	31	72.26 ± 19.01
Clinical success	31	25 (80.6)
Days from procedure to follow-up, median (IQR)	31	261.00 (170.50;417.50)
Months from procedure to follow-up, median (IQR)	31	8.56 (5.59;13.69)

SD—standard deviation, IQR—interquartile range. Numeric variables were presented as mean ± SD if the distribution was normal and median (IQR) if the distribution was not normal. * Maximum out of all procedures per patient. ** Hepatic artery dissection (*n* = 2) or legs tingling (*n* = 1). *** Mean of all procedures per patient.

**Table 2:** Comparison between procedures with and without clinical success.

Variable	Clinical Success		MD (95% CI)	*p*
	Yes (*n* = 25)	No (*n* = 6)		
Age (day of 1st procedure), years, mean ± SD	48.96 ± 11.48	51.15 ± 8.95	−2.19 (−12.49;8.12) ^1^	0.667 ^2^
Sex				
Female	22 (88.0)	3 (50.0)	-	0.069 ^3^
Male	3 (12.0)	3 (50.0)		
Location				
Left liver lobe	11 (44.0)	2 (33.3)	-	0.736 ^3^
Right liver lobe	13 (52.0)	4 (66.7)		
Both liver lobes	1 (4.0)	0 (0.0)		
Volume before procedure, cm^3^, median (IQR)	238.32 (139.23;534.75)	274.44 (141.81;484.51)	−36.12 (−255.93;293.71)	0.942
Embolization—no of stages				
1	9 (36.0)	5 (83.3)	-	0.154 ^3^
2	4 (16.0)	1 (16.7)		
3	10 (40.0)	0 (0.0)		
4	2 (8.0)	0 (0.0)		
Bleomycin, UI, median (IQR)	26,250.00 (15,000.00;30,000.00)	15,000.00 (15,000.00;15,000.00)	11,250.00 (0.00;15,000.00)	0.108
Lipiodol, mL, median (IQR)	0.00 (0.00;10.00)	2.50 (0.00;5.00)	−2.50 (−5.00;10.00)	0.666
Lipiodol > 0, mL, median (IQR)	15.75 (10.00;36.25)	5.00 (5.00;12.50)	10.75 (−10.00;35.00)	0.162
Radiation, Gy, median (IQR)	0.21 (0.00;0.60)	0.00 (0.00;0.17)	0.21 (0.00;0.58)	0.306
Radiation > 0, Gy, median (IQR)	0.58 (0.25;0.85)	0.67 (0.45;0.89)	−0.08 (−0.93;2.11)	>0.999
Coverage of hemangioma’s borders *				
<25%	1 (4.0)	0 (0.0)	-	>0.999 ^3^
25–50%	0 (0.0)	0 (0.0)		
50–75%	1 (4.0)	0 (0.0)		
75–100%	23 (92.0)	6 (100.0)		
Days from procedure to follow-up, median (IQR)	263.00 (180.00;453.00)	252.50 (169.75;351.75)	10.50 (−152.00;237.00)	0.789

SD—standard deviation, IQR—interquartile range, MD—mean ^1^ or median difference. Numeric variables were presented as mean ± SD if the distribution was normal and median (IQR) if the distribution was not normal. Groups compared with t-Student test ^2^, t-Welch test ^3^, Mann–Whitney U test, as appropriate. * Maximum out of all procedures per patient.

**Table 3:** Logistic regression outcomes for clinical success.

	Univariate Models			Multivariate Model		
Variable	OR	95% CI	*p*	OR	95% CI	*p*
Age (day of 1st procedure), years	0.98	0.90–1.07	0.655	-	-	-
Sex, male (vs. female)	0.14	0.02–1.01	0.051	0.08	0.00–1.03	0.072
Location						
Right liver lobe (vs. left liver lobe)	0.59	0.07–3.65	0.583	-	-	-
Both liver lobes (vs. left liver lobe)	Inf	-	0.995	-	-	-
Diagnostics before procedure, MR (vs. TK)	0.94	0.10–21.10	0.961	-	-	-
Size before procedure, cm						
x	1.05	0.79–1.52	0.747	-	-	-
y	1.05	0.81–1.48	0.750	-	-	-
z	1.01	0.80–1.37	0.916	-	-	-
Volume before procedure, cm^3^	1.00	1.00–1.00	0.673	-	-	-
Embolization—no of stages						
2 (vs. 1)	Inf	-	0.996	-	-	-
3 (vs. 1)	2.22	0.24–50.09	0.523	-	-	-
4 (vs. 1)	Inf	-	0.998	-	-	-
Embolization—no of stages	2.85	0.85–18.49	0.161	6.68	1.13–190.28	0.116
Bleomycin, UI	1.00	1.00–1.00	0.211	-	-	-
Lipiodol, mL	1.04	0.97–1.18	0.409	-	-	-
Lipiodol > 0, mL	1.10	0.98–1.41	0.278			
Radiation, Gy	2.34	0.49–53.03	0.450	-	-	-
Radiation > 0, Gy	1.24	0.22–44.67	0.849			
Coverage of hemangioma’s borders, 50–75% (vs. 75–100%) *	Inf	-	0.995			
Hepatic artery dissection (vs. no complication)	0.43	0.03–10.42	0.528	-	-	-
Post-embolization syndrome–severity [0–3] **	0.50	0.16–1.44	0.190	-	-	-
Post-embolization syndrome–severity > 0 [1–3] **	0.51	0.13–1.87	0.289	-	-	-
Post-embolization syndrome—vomiting	0.27	0.03–2.56	0.221	0.04	0.00–1.12	0.089
Hospitalization, days	0.98	0.85–1.19	0.829	-	-	-
Diagnostics after procedurę, MR (vs. TK)	Inf	-	0.995	-	-	-
Size after procedure, cm						
x	0.70	0.42–1.02	0.101	-	-	-
y	0.69	0.45–0.97	0.053	-	-	-
z	0.78	0.54–1.08	0.140	-	-	-
Volume after procedure, cm^3^	1.00	0.99–1.00	0.206	-	-	-
Days from procedurę to follow-up	1.00	1.00–1.00	0.610	-	-	-
Months from procedurę to follow-up	1.02	0.95–1.16	0.610	-	-	-

OR—odds ratio, CI—confidence interval. * Maximum out of all procedures per patient. ** Mean of all procedures per patient.

**Table 4:** Relation between volume regression and coverage of hemangioma’s borders.

Variable	*n*	Volume Regression, %	MD (95% CI)	*p*
Coverage of hemangioma’s borders (1st procedure)				
<75%	8	78.12 ± 16.56	7.91 (−8.05;23.86)	0.319
75–100%	23	70.22 ± 19.72		
Coverage of hemangioma’s borders (maximum of procedures per patient)				
<75%	2	77.00 (72.97;81.04)	3.32 (−23.64;39.88) ^1^	0.968 ^2^
75–100%	29	73.68 (63.06;91.08)		

Data presented as mean ± standard deviation or median (interquartile range) ^1^, depending on normality of distribution. MD—mean or median difference, CI—confidence interval. Groups compared with t-Student test or Mann–Whitney U test ^2^.

**Table 5:** Relation between volume regression and location of the change.

Variable	*n*	Volume Regression, %	MD (95% CI)	*p*
Location				
Left liver lobe	13	71.68 ± 18.05	0.15 (−14.42;14.72)	0.986
Right liver lobe	17	71.54 ± 20.20		

Data presented as mean ± standard deviation. MD—mean difference, CI—confidence interval. Groups compared with *t*-Student test.

**Table 6:** Relation between vomiting (post-embolization syndrome) and bleomycin dose.

Variable	*n*	Bleomycin, UI	MD (95% CI)	*p*
Post-embolization syndrome—vomiting				
Yes	5	30,000.00 (26,250.00;37,500.00)	13,000.00 (−3750.00;20,000.00)	0.181
No	26	17,000.00 (15,000.00;30,000.00)		

Data presented as median (interquartile range). MD—median difference, CI—confidence interval. Groups compared with Mann–Whitney U test.

**Table 7:** Relation between vomiting (post-embolization syndrome) and lipiodol dose.

Variable	*n*	Lipiodol, mL	MD (95% CI)	*p*
Post-embolization syndrome—vomiting				
Yes	3	21.50 (20.75;22.00)	11.50 (−17.00;20.50)	0.418
No	12	10.00 (8.75;36.25)		

Data presented as median (interquartile range). MD—median difference, CI—confidence interval. Groups compared with Mann–Whitney U test.

**Table 8:** Relation between coverage of hemangioma’s borders and post-embolization syndrome.

Variable	Coverage of Hemangioma’s Borders		MD (95% CI)	*p*
	<75% (*n* = 2)	75–100% (*n* = 29)		
Post-embolization syndrome—severity, [0–3]	0.75 (0.38;1.12)	0.50 (0.00;1.00)	0.25 (−1.50;1.50)	>0.999
Post-embolization syndrome—vomiting	0 (0.0)	5 (17.2)	-	>0.999 ^1^

Data presented as median (interquartile range) for severity and *n* (% of group) for vomiting. MD—median difference, CI—confidence interval. Groups compared with Mann–Whitney U test or Fisher exact test ^1^.

## Data Availability

Data available on request.
